# Case of a Succession of Monstrous Births Occurring in the Same Female

**Published:** 1849-01

**Authors:** 


*Case of a succession of monstrous births occurring in the same female. Extract of a letter to Professor* DUNGLISON, *from* DR. J. MARTIN, *dated*

PHILADELPHIA, DEC. 4TH, 1848.

I will cite the cases of monstrosity occurring in a lady with whom I have been well acquainted for about fourteen years. Previous to that time she gave birth to two well formed and well featured children. I attended her in the first case of monstrosity. At the period of my earliest acquaintance with her, she moved in the middle walks of society, and had enjoyed good health up to that time. She was well developed in figure, and gave birth to a child perfectly formed in every respect with the exception of its head and face. The eyes were placed at the top of the forehead, and all the superior and posterior parts of the head were deficient, the corresponding bones of the cranium being wanting, and the opening fringed round with something very like liver. Her labour was attended with no difficulty; but the liquor amnii was very abundant, and the child was still-born. She had a speedy recovery.

In about eighteen months after this she gave birth to another child which was properly developed as to face and head, but the flexor muscles of the legs and arms were in fault; I could not straighten its legs, arms, fingers, or toes, owing to the flexors being too short. Her labour, this time, was not so easy, in consequence of the arms being flexed on the breast, and the hands below the chin; yet it was not attended with much difficulty, and the liquor amnii was again abundant.

The next two confinements were similar to the first I have detailed, in every respect, the monstrosity being the same; from both she had a speedy recovery.

At the next, (in the winter of 1843–4,) I was not present, being in attendance on the medical lectures in Philadelphia, but I understood that the labour was not accompanied with more severity than is common to woman; the child was defective on the top of the head, and the liver-like growth was there as in the other cases.

In 1846 she was confined again, and I was summoned to watch over her difficulties during her labour. She was in great hope that she would have a perfect child, being led to this conclusion by the strong movements of the child *in utero.* During the labour the os tincæ dilated rather reluctantly; but when dilatation did occur and the membranes were ruptured, a great discharge of liquor amnii took place, and I was enabled to discover that the head was defective while it was still at the superior strait. The deficiency was the same as in the other instances, but it was born alive and lived about three hours, and moved and made considerable muscular exertion. There was hemorrhage from the liver-like production on the top of the head. This child assumed a cerulean hue, becoming more livid from the moment of its birth till its death. The lady recovered rapidly.

The last misfortune was in 1847, when she had a miscarriage; the embryo was about two inches in length, and the defect at the top of the head could be very readily discovered.

Here then, are five cases of mature labour, the products of which were all defective at the top of the cranium. One carried to the full term and defective in the flexor muscles, and one abortion, in which the embryo exhibited the same defect at the upper portion of the cranium, and all occurring in the same female apparently in the enjoyment of perfect health.

